# Oxidative Stress and Effect of Treatment on the Oxidation Product Decomposition Processes in IBD

**DOI:** 10.1155/2018/7918261

**Published:** 2018-07-02

**Authors:** Ewa Dudzińska, Magdalena Gryzinska, Katarzyna Ognik, Paulina Gil-Kulik, Janusz Kocki

**Affiliations:** ^1^Chair of Public Health, Medical University of Lublin, 20-093 Lublin, Poland; ^2^Institute of Biological Basis of Animal Production, Subdepartment of General and Molecular Genetics, University of Life Sciences in Lublin, 20-950 Lublin, Poland; ^3^Department of Biochemistry and Toxicology, University of Life Sciences in Lublin, 20-950 Lublin, Poland; ^4^Chair of Medical Genetics, Department of Clinical Genetics, Medical University of Lublin, 20-080 Lublin, Poland

## Abstract

Oxidative stress plays an important role in IBD because chronic intestinal inflammation is associated with the overproduction of reactive oxygen species (ROS) leading to oxidative stress, which has been implicated in IBD. Many lines of evidence suggest that IBD is associated with an imbalance between ROS and antioxidant activity which generates oxidative stress as the result of either ROS overproduction or a decrease in antioxidant activity. Our study was to evaluate the influence of oxidative stress and antioxidants on the course of the disease and treatment of IBD patients. Our results show that an increase of LOOH levels positively correlates with an increase in MDA levels; therefore, MDA may be a marker indicating lipid peroxidation. Also, being the decomposition product of oxidation processes, MDA may be applied as a useful biomarker for identifying the effect of endogenous oxidative stress in Crohn's disease patients. The anti-inflammatory efficacy of AZA drugs may be the result of a reduction of the amount of lipid peroxides in the intestinal mucosa cells in CD patients and facilitate mucosal healing.

## 1. Introduction

Inflammatory bowel diseases (IBDs) mainly include Crohn's disease (CD) and ulcerative colitis (UC). Both conditions constitute a chronic and relapsing disorder of the gastrointestinal tract (GI), associated with an exacerbated intestinal immune response to harmless stimulus, leading to an upregulation of proinflammatory mediators, which may trigger the onset and perpetuation of IBD [1–3]. Despite some overlapping clinical features, these diseases are defined by separate inflammatory profiles, gut microbiota composition, and symptomatology. CD affects any portion of the alimentary tract and is defined by a discontinuous and ulcerous transmural inflammation, associated with intestinal granulomas, obstructions, abscesses, strictures, and fistulas. In UC, inflammation involves only the superficial layers of the intestinal mucosa and is localized to regions of the gut most highly colonized by bacteria, specifically at the rectum and moving proximally along the large bowel [4]. The accumulation of ROS could cause damage to specific genes involved in cell growth or differentiation and could cause changes in antioxidant enzyme levels. Oxidative stress in IBD patients with increased ROS levels and decreased antioxidant levels in the inflamed mucosa, could ultimately contribute to chronic tissue damage [5]. ROS is the mediator responsible for the intracellular damage of lipids, proteins, carbohydrates, and nucleic acids and are highly reactive due to their unstable conditions with unpaired electrons [6, 7]. RON causes intestinal tissue lipid peroxidation and, consequently, disruption of intercellular junctions, as well as leukocyte and neutrophil infiltration that produces ROS, and cytokines that lead to the inflammatory process [8]. Moreover, ROS upregulates the expression of genes involved in adaptive and innate immune responses in the GI tract [7]. Increased ROS, which is produced through the oxidative stress associated with the inflammatory process in cells, may lead to severe damage to macromolecules [5].

Once initiated by any of several pathways, lipid peroxidation, the oxidative damage of membrane lipids, spreads aggressively in a self-propagating chain reaction and intensifies oxidative damage. Lipid peroxides adversely change membrane structure and function and generate highly reactive toxic secondary products that react with DNA and proteins, compromising normal activity [9]. Defense mechanisms minimize the toxic effects of these compounds but in some cases are insufficient and lead to initiation and progression of many diseases such as IBD [6]. Among the antioxidant enzymes, the catalyzed superoxide dismutase (SOD) is a mutation of O_2_ to H_2_O_2_ and molecular oxygen, while the decomposition of H_2_O_2_ to nontoxic compounds is the main function of catalase (CAT), peroxiredoxins, and glutathione peroxidase (GPx) [10]. The functioning of the antioxidant system plays an important role in elements such as zinc, which plays a pivotal role in wound repair, tissue regeneration, and the immune response [11]. Zinc excess induces copper deficiency, which has been related to multiple adverse effects, such as decreased expression of copper-dependent enzymes (e.g., superoxide dismutase and ceruloplasmin), which are important in antioxidant defense [12]. Although the role of oxidative stress and antioxidants in IBD has been evaluated, their role in the pathogenesis of IBD is not well understood.

The present study was undertaken mainly to evaluate the influence of oxidative stress and antioxidants on the course of the disease and on the treatment of IBD patients.

## 2. Material and Methods

A combined group of 90 individuals took part in the study, including 40 IBD patients and 50 healthy individuals. The study was carried out in the years 2014–2016 among patients of the gastroenterology ward and gastroenterology clinic of the Cardinal Stefan Wyszyński Regional Specialist Hospital in Lublin. A group of 20 individuals, comprised patients diagnosed with Crohn's disease (CD), K50 according to the ICD-10 criteria. The age range in this group was 19–52 years, and the mean age was 33. A group of 20 individuals comprised patients diagnosed with ulcerative colitis, K51 according to the ICD-10 classification (UC). The age range in this group was 21–78 years, with a mean age of 37 years 6 months. The control consisted of 50 healthy volunteers (controls). This group was aged 19–67, with a mean age of 40 years. The material for the study consisted of plasma isolated from peripheral blood collected from the 40 IBD patients and the 50 healthy individuals (blood was collected once).

Concentrations of lipid hydroperoxides (LOOH) and malondialdehyde (MDA) were determined using kits produced by Cell Biolabs Inc., San Diego, USA. A diagnostic kit manufactured by Oxis International Inc., Portland, USA, was used to determine the activity of superoxide dismutase (SOD) and catalase (CAT). In blood plasma, the activity of ceruloplasmin (Cp) was determined using the Ceruloplasmin ELISA kit (Biomatik, Delaware, USA) and total glutathione was determined (GSH + GSSG) using the Total Glutathione Assay (Cell Biolabs Inc., San Diego, USA). The content of Cu and Zn in the samples of blood was determined by inductively coupled plasma optical emission spectrometry (ICP-OES).

## 3. Results

Table 1shows the results of Cu, Zn, Na, CAT, GSH + GSSG, MDA, LOOH, and Cp levels among CD and UC patients and control individuals, which we compare against Table 2 showing the results of the correlation of age, duration, CRP, WBC, RBC Cu, Zn, Na, CAT, GSH + GSSG, MDA, LOOH, and Cp among CD and UC patients and control individuals (Pearson correlation coefficient).

**Figure 1:**
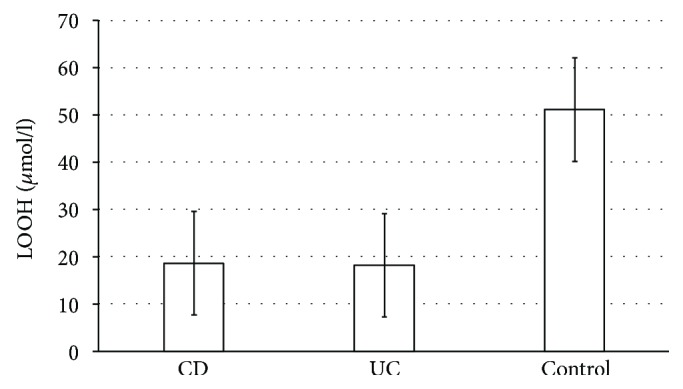
The mean values of LOOH concentrations (*μ*mol/l) depending on the study group (UC, CD, and control group).

The mean values of LOOH concentrations (*μ*mol/l) in the plasma of patients with CD (18.52 ± 10.99 *μ*mol/l) and UC (18.06 ± 8 *μ*mol/l) is significantly lower (*p* < 0.001) compared to that of the control group of healthy people (51.08 ± 20.58 *μ*mol/l) (Figure 1).

**Figure 2:**
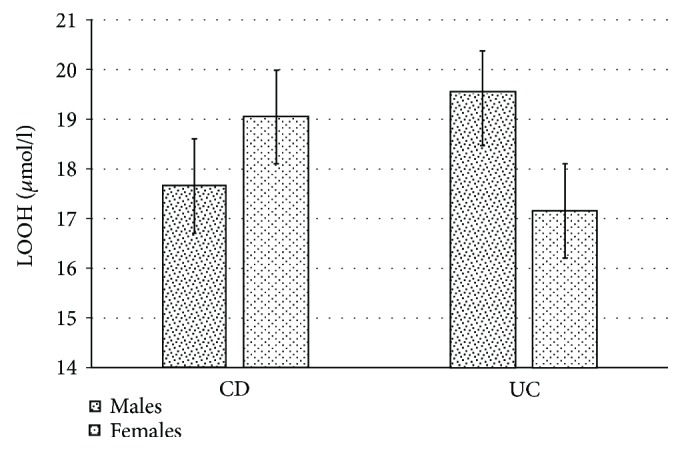
Differences in LOOH (*μ*mol/l) concentrations in the plasma of patients with CD and UC depending on gender.

It was shown that the mean LOOH concentration (*μ*mol/l) in women with UC (14.61 ± 4.94 *μ*mol/l) is significantly lower (*p* = 0.043) in comparison to that in men with UC (20.12 ± 5.71 *μ*mol/l) (Figure 2). In patients with CD, no significant difference in LOOH concentration between the sexes was found (Figure 2).

**Figure 3:**
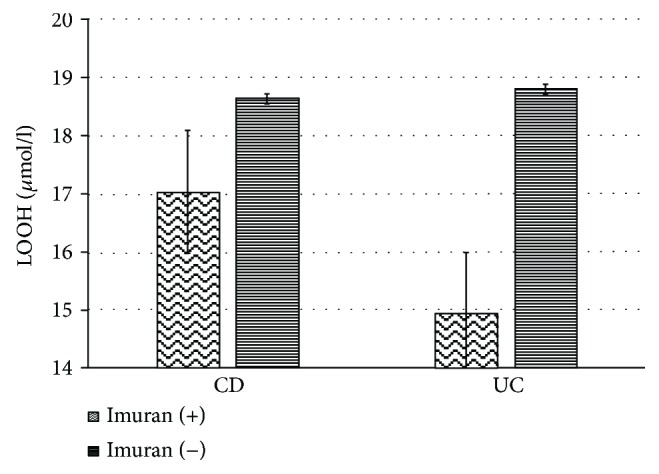
Differences in the concentration of LOOH (*μ*mol/l) in the blood plasma of CD patients taking Imuran and those with CD and UC who did not take Imuran.

It has been shown that in patients taking Imuran in CD, the mean LOOH (9.91 ± 4.11 mmol/l) is significantly lower (*p* = 0.007) compared to that in CD patients not taking this drug (18.62 ± 6.69) (Figure 3).

**Figure 4:**
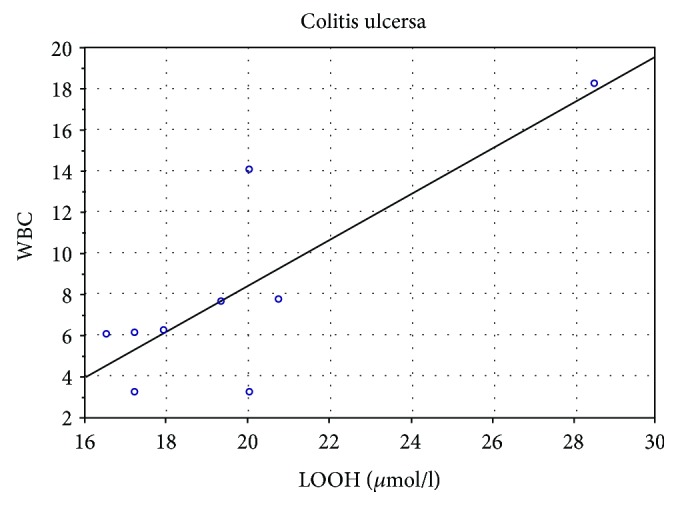
Correlation of LOOH (*μ*mol/l) with the level of leukocytes in patients with UC.

Figure 4 shows the positive correlation of LOOH concentration (*μ*mol/l) with leukocyte level (*r* = 0.89*p* < 0.05) in patients with UC.

**Figure 5:**
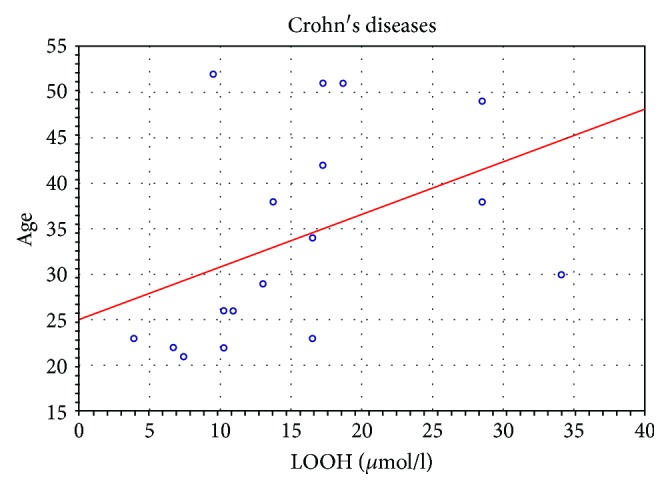
Correlation of LOOH (*μ*mol/l) with age in patients with CD.

Patients with CD showed a positive correlation of LOOH concentration with age (*r* = 0.79, *p* < 0.05) (Figure 5).

**Figure 6:**
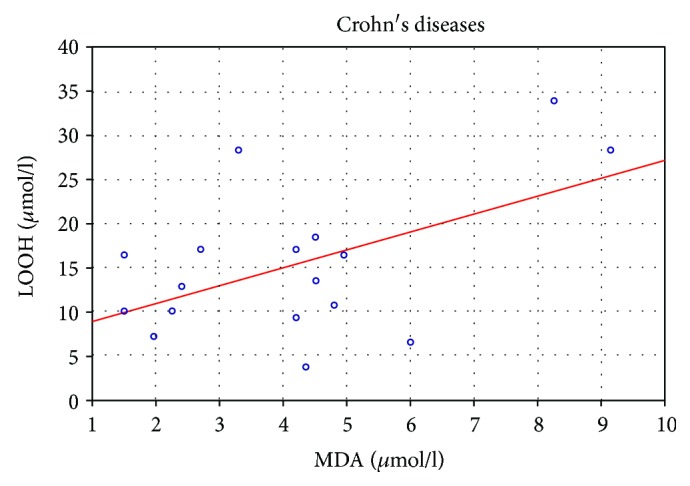
Correlation of LOOH (*μ*mol/l) with MDA (*μ*mol/l) in CD patients.

Patients with CD showed a positive correlation between LOOH concentration and MDA concentration (*r* = 0.52, *p* < 0.05) (Figure 6).

## 4. Discussion

The gastrointestinal tract is a major site for the generation of prooxidants, whose production is primarily due to the presence of a variety of microbes, food ingredients, and interactions between immune cells. To eliminate ROS, intestinal cells have several enzymatic and nonenzymatic antioxidants, including SOD, GSH, and CAT, but excessive generation of ROS enhances LP and could deplete antioxidant defenses [8]. The susceptibility of a given organ to oxidative stress depends on its antioxidant defense status, and a general balance between oxidants and antioxidants is required to maintain cellular homeostasis [9].

Several lines of evidence suggest that regarding trace elements with antioxidant functions, reduced plasma, selenium, and zinc levels were reported in IBD patients as compared to that in healthy controls [13]. Other lines of evidence indicate that copper concentrations did not differ between patients with IBD and controls [14]. This may suggest different antioxidative mechanisms in patients with IBD.

Our studies have shown that levels of antioxidants such as Zn, Cu, SOD, CAT, GSH + GSSG, and Cp in IBD patients as compared to that in healthy controls do not show significant differences (Table 1). Discrepancies concerning the plasma activity of the enzymes may result from applied treatment derivatives of 5-ASA, sulfasalazine, and mesalazine. Previous investigations reported that sulfasalazine decreased ROS concentration. It has been pointed out that inflammation has been extensively associated with an increase in oxidative stress; the protection afforded by 5-ASA could be related to their well-known antioxidant properties [15]. It was shown that UC patients who were taking 5-ASA, besides having the lowest number of flares per year of follow-up, also exhibited the lowest level of DNA damage. Moreover, the value found was even lower than the DNA damage found in controls, and this could be attributed to the antioxidant and free radical scavenging action of 5-ASA [16]. In the UC process, the antioxidant capacity of the damaged mucosa is compromised. Therefore, the use of nontraditional therapy that eliminates ROS, inhibits cell damage, and improves the activity of antioxidant enzymes, can be beneficial, either associated or not with anti-inflammatory medicines [8]. Significantly lower LOOH in women than in men may suggest that women take more antioxidants compared to men. This is in agreement with our report where we showed that women with CD showed a significantly higher level of Zn (*μ*mol/l) (*p* = 0.008) and Cu (*μ*mol/l) (*p* = 0.007) in comparison with men with CD (Table 1). Balmus et al. [17] noted that an excess of lipid peroxidation is probably an important pathogenetic factor in IBDs. We, in turn demonstrated that the levels of plasma LOOH were diminished in IBD patients regardless of disease type as compared to that in healthy controls (Figure 1). Our results suggest that the effect of the treatment applied has an impact on reducing lipid peroxidation and reduction of LOOH.

In this study, the measurement of lipid peroxidation showed a decrease in the level of LOOH markers in UC and CD patients taking Imuran compared to the patients not taking this drug (Figure 3). Azathioprine (AZA) is an immune response modifier drug used in patients with reduced 5-aminosalicylate response. AZA is a prodrug that is converted into the body to 6-mercaptopurine, an active metabolite effective for inflammatory bowel disease [18]. AZA induces apoptosis in activated lymphocytes and these effects should be crucial in determining the efficacy of these medications as immunomodulators in patients with IBD [19]. AZA is a purine analog inhibiting the synthesis of purine nucleotide and is frequently associated with a number of adverse effects; for this reason, it is has been discontinued [20]. These adverse effects include pancreatitis, fever, rash, malaise, nausea, diarrhea, hepatitis, leukopenia, and some forms of hepatitis [20].

The study of Sood et al. [21] has been shown that AZA is an established treatment for UC patients. The authors studied the effectiveness of AZA in a large cohort of UC (225) patients, of whom 154 achieved sustained clinical benefit. This shows that AZA is a safe and effective therapy in UC patients who fail 5-aminosalicylates.

In a review study by Axelrad et al. [22], the authors concluded that AZA is more effective than placebo for the maintenance of remission in CD and there are several randomized controlled studies regarding the efficacy of thiopurines in inducing remission in CD. Therefore, AZA is widely used in the treatment of IBD. This drug can induce and maintain remission with a response rate of 55% to 70% [20]. AZA is an efficient anti-inflammatory drug that decreases infiltration of inflammatory cells into the ileal mucosa in CD patients and facilitates mucosal healing [15]. Our research may suggest that AZA may have an impact on the decrease of lipid peroxides responsible for many of the damaging reactions in the cell membranes. Moreover in our study, we have demonstrated that LOOH (*μ*mol/l) correlates positively with age (Figure 5).

The peroxidation of lipids can result in damage to the cell membrane due to the high concentration of lipids present. The end products of lipid peroxidation can be both mutagenic and carcinogenic and can play a role in aging and disease progression [23]. There is a growing amount of experimental evidence proving the free radical theory of aging. The formation of reactive oxygen species increases with age, ultimately resulting in damage to cells and their components such as proteins, lipids, and DNA [24].

A major hallmark of aging, and a key driver for the onset of age-related pathophysiologies is the disruption of cellular redox homeostatic mechanisms that protect against environmental factors, such as oxidative, pathological, and toxicological insults [25]. During aging, weakened antioxidant defenses allow the accumulation of toxic reactive oxygen species that contribute to aging and to the onset of multiple diseases [9].

In studies concerning effect of exercise-induced oxidative stress on health and aging, it was shown that lipid peroxidation increased significantly with age and submaximal effort [26]. Also, researchers Chen et al. [9] emphasize that lipid peroxides are generated by oxidative stress in cells and contribute to ageing. Furthermore, our studies have shown a positive correlation between LOOH and the level of leukocytes in UC patients (Figure 5).

Research stressed that infiltration of the mucosa by T cells and the increased production of proinflammatory T cell cytokines are a hallmark of IBD [27]. Bisping et al. [28] showed that intestinal epithelial cells (iEC) play a key role in the initiation and perpetuation of intestinal inflammation, and T cell/monocyte interactions influence epithelial physiology in states of inflammation. Research shows that T cells and T cell-derived cytokines induce and regulate mucosal immune response stimulation of epithelial proliferation and modulation of epithelial secretory activities. It was also demonstrated that iEC influences T lymphocytes as they downregulate intraepithelial lymphocytes. In CD and UC, many immunoregulatory abnormalities in intestinal and peripheral T lymphocytes are noted, for example, the altered ratio of proinflammatory to immunosuppressive cytokines or the selective activation of T-helper lymphocyte subsets and abnormalities in epithelial antigen presentation.

Additionally in studies on colonic biopsies, Balmus et al. showed that oxidative stress could be correlated to disease activity and the main sites of ROS production are phagocytic leukocytes during their massive infiltration of the intestinal mucosa in inflammation [17]. C-reactive protein (CRP) is a valuable marker to detect and follow-up disease activity in CD. UC has only a modest to absent CRP response despite active inflammation, and the reason for this is unknown [29, 30]. Our data showed that LOOH is negatively correlated with CRP in CD patients (Table 2).

In their studies, Pinto et al. [30] described a decreased plasmatic and erythrocyte glutathione peroxidase (GPx) activity in remission with a subsequent increase in active inflammation. This modulation suggests a compensatory role of GPx in the oxidative stress caused by active bowel inflammation in CD. These studies indicate the defense of the cell against inflammation by the growth of antioxidants. That is why negative correlation between LOOH and CRP may suggest cell defense against apoptosis. In the present study, it has been shown that elevated plasma lipid peroxide products are associated with elevated malondialdehyde (MDA) levels in CD patients (Figure 5). Lipid peroxidation was documented by higher plasma MDA concentrations in CD patients [31].

In this research, increased levels of lipid peroxides which are secreted into the blood circulation from the gut of CD patients to produce systemic effects have been pointed out. MDA was significantly increased during both active and inactive phases, though the second group tended to harbour lower levels [32]. The decomposition products of lipid peroxidation, such as MDA, can affect membrane proteins by cross-linkage, rendering them useless as receptors or enzymes. Lipid peroxide can lead to cellular dysfunction and may result in the deaths of the affected cells [33]. MDA is the final result of lipoperoxidation and is used as a marker of lipid peroxidation. It was estimated to indicate peroxide damage to membranes caused by inflammation. MDA levels in ileal segments from the colitic rats were higher as compared to those in the control rats [34]. Our results show that the increase of LOOH positively correlates with MDA, therefore MDA may be a marker indicating lipid peroxidation.

## 5. Conclusions

We arrive at the following conclusions:

1. The decomposition products of oxidation processes, MDA, may be applied as a useful biomarker for identifying the effect of endogenous oxidative stress in Crohn's disease patients.
2. The anti-inflammatory efficacy of AZA drugs may be the result of a reduction in the amount of lipid peroxides into the intestinal mucosa cells in CD patients and facilitate mucosal healing.

## Figures and Tables

**Table 1:** Levels of Cu, Zn, Na, CAT, GSH + GSSG, MDA, LOOH, and Cp among CD and UC patients and control individuals (mean ± SD).

Parameters	Control *n* = 50	CD *n* = 20	UC *n* = 20
Cu (*μ*mol/l)	17.44 ± 4.05	12.46 ± 11.59	12.18 ± 7.59
Zn (*μ*mol/l)	13.82 ± 2.77	19.61 ± 12.65	21.37 ± 8.52
SOD (U/l)	51,172.16 ± 23,136.88	51,610.23 ± 11,519.05	80,331.09 ± 35,002.31
CAT (U/l)	94,452.77 ± 169,943.87	45,030.43 ± 25,470.33	120,306.51 ± 158,262.62
GSH + GSSG (*μ*mol/l)	0.70 ± 0.49	1.04 ± 0.90	0.77 ± 0.62
MDA (*μ*mol/l)	6.72 ± 2.21	4.15 ± 2.16	3.87 ± 2.32
LOOH (*μ*mol/l)	51.08 ± 20.58	15.44 ± 8.31	17.06 ± 5.85
Cp (*μ*mol/l)	212.84 ± 17.73	199.09 ± 85.67	234.40 ± 76.98

**Table 2:** Correlation of age, duration, CRP, WBC, RBC Cu, Zn, Na, CAT, GSH + GSSG, MDA, LOOH, and Cp among CD and UC patients and control individuals.

Parameters	Age	Duration	CRP	WBC	RBC	Cu (*μ*mol/l)	Zn (*μ*mol/l)	SOD (U/l)	CAT (U/l)	GSH + GSSG (*μ*mol/l)	MDA (*μ*mol/l)	LOOH (*μ*mol/l)	Cp (*μ*mol/l)
Condition	UC												
Cu (*μ*mol/l)	0.536	0.303	−0.216	−0.198	0.605	—	0.652	0.549	0.382	−0.190	−0.044	−0.009	0.766^∗^
Zn (*μ*mol/l)	−0.155	−0.200	−0.228	−0.636	0.266	0.652	—	0.740	0.422	0.445	0.520	−0.492	0.898^∗^
SOD (U/l)	0.260	0.172	−0.257	−0.351	−0.063	0.549	0.740	—	−0.024	0.241	0.079	−0.364	0.510
CAT (U/l)	−0.316	−0.683	−0.234	−0.547	0.513	0.382	0.422	−0.024	—	−0.152	0.531	−0.464	0.697
GSH + GSSG (*μ*mol/l)	−0.516	−0.261	0.497	−0.313	−0.569	−0.190	0.445	0.241	−0.152	—	0.698	−0.101	0.257
MDA (*μ*mol/l)	−0.652	−0.679	0.202	−0.751	−0.214	−0.044	0.520	0.079	0.531	0.698	—	−0.510	0.583
LOOH (*μ*mol/l)	0.439	0.486	0.692	0.886^∗^	−0.151	−0.009	−0.492	−0.364	−0.464	−0.101	−0.510	—	−0.409
Cp (*μ*mol/l)	−0.072	−0.271	−0.152	−0.651	0.431	0.766^∗^	0.898^∗^	0.510	0.697	0.257	0.583	−0.409	—

Condition	CD												
Cu (*μ*mol/l)	−0.110	−0.528	0.855^∗^	0.251	0.022	—	0.686^∗^	0.797^∗^	−0.048	0.369	−0.692^∗^	0.219	0.715^∗^
Zn (*μ*mol/l)	0.015	−0.693^∗^	0.423	−0.046	0.030	0.686^∗^	—	0.554	0.236	0.177	−0.273	0.192	0.598
SOD (U/l)	−0.198	−0.491	0.698^∗^	0.113	−0.214	0.797^∗^	0.554	—	0.041	−0.106	−0.721^∗^	0.280	0.581
CAT (U/l)	−0.168	−0.250	0.138	0.001	−0.073	−0.048	0.236	0.041	—	−0.167	0.070	−0.007	0.127
GSH + GSSG (*μ*mol/l)	−0.214	−0.274	0.258	−0.272	−0.195	0.369	0.177	−0.106	−0.167	—	0.196	−0.446	0.008
MDA (*μ*mol/l)	0.043	0.127	−0.632^∗^	−0.471	−0.210	−0.692^∗^	−0.273	−0.721^∗^	0.070	0.196	—	−0.355	−0.386^∗^
LOOH (*μ*mol/l)	0.792^∗^	0.299	0.084	0.320	0.627	0.219	0.192	0.280	−0.007	−0.446	−0.355	—	0.299
Cp (*μ*mol/l)	0.117	−0.456	0.604	0.219	0.137	0.715^∗^	0.598	0.581	0.127	0.008	0.528^∗^	0.299	—

^∗^ *p*<0.05.

## Data Availability

The data used to support the findings of this study are included within the article.
